# Exploration of age-related mitochondrial dysfunction and the anti-aging effects of resveratrol in zebrafish retina

**DOI:** 10.18632/aging.101966

**Published:** 2019-05-19

**Authors:** Ning Wang, Zhiwen Luo, Ming Jin, Weiwei Sheng, Han-Tsing Wang, Xinyi Long, Yue Wu, Piaopiao Hu, Hong Xu, Xu Zhang

**Affiliations:** 1 Affiliated Eye Hospital of Nanchang University, Jiangxi Research Institute of Ophthalmology and Visual Science, Nanchang, China; 2 Queen Mary School of Nanchang University, Nanchang, China; 3 Institute of Life Science of Nanchang University, Nanchang, China; 4 School of Life Sciences of Nanchang University, Nanchang, China; 5 Jiangxi Provincial Collaborative Innovation Center for Cardiovascular, Digestive and Neuropsychiatric Diseases, Nanchang, China; * Equal contribution

**Keywords:** aging zebrafish, retina, mitochondrial dysfunction, mitophagy, mTOR, resveratrol

## Abstract

It is currently believed that aging is closely linked with mitochondrial dysfunction, and that resveratrol exhibits anti-aging and neuroprotective effects by improving mitochondrial function, even though the mechanisms are not well defined. This study explored mitochondrial quality (mitochondrial DNA integrity and copy number), mitochondrial function (fusion/fission, mitophagy/autophagy), antioxidant system and activity of the Akt/mTOR and Ampk/Sirt1/Pgc1α pathways, and inflammation in aging zebrafish retinas to identify the probable mechanisms of resveratrol’s anti-aging and neuroprotective effects. mtDNA integrity, mtDNA copy number, mitochondrial fusion regulators, mitophagy, and antioxidant-related genes were all decreased whereas Akt/mTOR activity and inflammation was increased upon aging in zebrafish retinas. Resveratrol was shown to not only increase mitochondrial quality and function, but also to suppress Akt/mTOR activity in zebrafish retinas. These results support the notion that mitochondrial dysfunction and increased Akt/mTOR activity are major players in age-related retinal neuropathy in zebrafish, and demonstrate a trend towards mitochondrial fragmentation in the aging retina. Importantly, resveratrol promoted mitochondrial function, up-regulating Ampk/Sirt1/Pgc1α, and down-regulated Akt/mTOR pathway activity in zebrafish retinas, suggesting that it may be able to prevent age-related oculopathy.

## Introduction

Aging is the biological process characterized by the accumulation of damage in structure and decline in function of cells and tissues over time, ultimately leading to organismal death [1]. The causes of aging are complex but include abnormal mitochondria, epigenetic alterations, increased reactive oxygen species (ROS), increased DNA methylation, and decreased telomere length [2–4]. Recently, both dysfunctional mitochondria that overproduce ROS [5] and abnormal mitochondrial dynamics have been recognized as crucial contributors to the aging process as well as age-related neuronal diseases and age-related oculopathies such as glaucoma, age-related macular degeneration (AMD), and cataracts [6–8]. The detailed mechanism by which dysfunctional mitochondria influence the aging process, however, is complex and incompletely understood.

Mitochondrial dysfunction includes defective mitochondrial fusion/fission, decreased mitochondrial DNA (mtDNA) quality, and altered mitophagy [9]. Mitochondrial fusion/fission determines the mitochondrial mass and network structure in the cell. Mitochondrial fusion is the process of joining multiple mitochondria together and is mediated by optic atrophy 1 (Opa1) and mitochondrial fusion protein 1 and 2 (Mfn1 and Mfn2). Mitochondrial fission, on the other hand, divides a single large mitochondrion into multiple smaller mitochondria, which is mediated by dynamin related protein 1 (Drp1) and mitochondrial fission protein 1 (Fis1) [10]. Some studies have found that upon aging, mitochondria tend to be more fragmented, suggesting that fusion is decreased and/or fission is increased. A potential reason for this alteration in mitochondrial dynamics is that accumulated ROS damage reduces mitochondrial output, and in response the cell promotes mitochondrial fission to help cope with the decline in mitochondrial function [11].

The integrity of mtDNA, which is highly vulnerable to ROS damage, is a good indicator of mitochondrial quality, and defective mtDNA is ubiquitous in aged tissues [12]. Mitochondrial fusion/fission plays an important role in mitigating the effects of mtDNA damage, and its breakdown can exacerbate the effects of aging. Fusion can help relieve mtDNA damage by diluting mutant mtDNA with non-mutant mtDNA, whereas fission can allow for turnover of mutant mtDNA through mitophagy [13]. On the other hand, mitochondrial fragmentation is associated with apoptosis and cell death rather than mitophagy and must be distinguished from fission [14].

Autophagy is the process by which cells degrade damaged organelles and other cellular materials and recycle cellular building blocks such as amino acids [15]. A significant decline in autophagy is found in aging, and many studies have shown that increased autophagy can extend organismal lifespan [16,17]. Notably, centenarians have been found to retain active autophagy [18]. Mitophagy, mediated by PTEN-induced putative kinase 1 (Pink1), is the highly selective autophagic process by which cells eliminate damaged mitochondria [19]. It is believed that mtDNA repair mechanisms are less efficient than those for nuclear DNA repair, despite mtDNA being much more prone to damage particularly by ROS. Mitophagy is therefore crucial for normal mitochondrial function by clearing damaged mtDNA. Similar to general autophagy, functional mitophagy has also been found to be decreased in aged cells including skin fibroblasts, muscle satellite cells, and neural cells [20–22]. Downregulation of mitophagy also has a strong correlation with neurodegenerative disease [23]. It is therefore believed that a decline in mitophagy leads to higher oxidative stress, lower quality mitochondria, and apoptosis, which ultimately accelerates aging [24].

Resveratrol, a plant natural product found in high levels in peanuts and grape skin, has well-established antioxidant, anti-inflammatory, anti-mutagenic, neuroprotective, and anti-aging effects in many species [25–27]. Current evidence suggests that the anti-aging effects of resveratrol are related to its ability to modulate mitochondria [28,29]. Resveratrol has been found to increase mitochondrial fusion/fission as well as promote Pink1 expression and autophagic activity [30–33]. Additionally, some reports indicate these effects of resveratrol are due to its ability to decrease mammalian target of rapamycin (mTOR) levels and promote Ampk/Sirt1 activities [9,34]. In a prior study, we have demonstrated that resveratrol can protect against retinal neuron degeneration through activation of SIRT1 and inhibition of the mTOR pathway and inflammation-related proteins; however, the full effects of resveratrol on mitochondria remains unclear [30,35,36].

Zebrafish has become a powerful and widely used model organism as they are easy and cheap to maintain, are sensitive to neurotropic drugs, and a single pair can spawn 200-300 eggs in one week who grow to sexual maturity in only 3-4 months [37,38]. 70% of zebrafish genes have homologs in humans, 84% of human disease-related genes have homologs in zebrafish, and CNS protein-encoding genes have very similar functions with their human counterparts [39]. Moreover, the human retina is more closely related to the zebrafish retina than that of mice, which has rod-dominant vision unlike the cone-dominant vision found in humans and zebrafish [37]. Zebrafish is also an excellent aging model owing to its short lifespan, gradual aging progression as in mammals, and similar aging markers to those in humans [40].

The aim of the current study was to investigate mitochondrial DNA quality, mitochondrial function, AKT/mTOR, antioxidant system and Ampk/Sirt1/Pgc1α activities in young and aging zebrafish retinas, and to analyze the anti-aging effect of resveratrol on these in the zebrafish retina. A mechanism related to mitochondrial quality control was investigated.

## RESULTS

### Zebrafish retinal mtDNA quality at different ages and after resveratrol treatment

**Figure 1:**
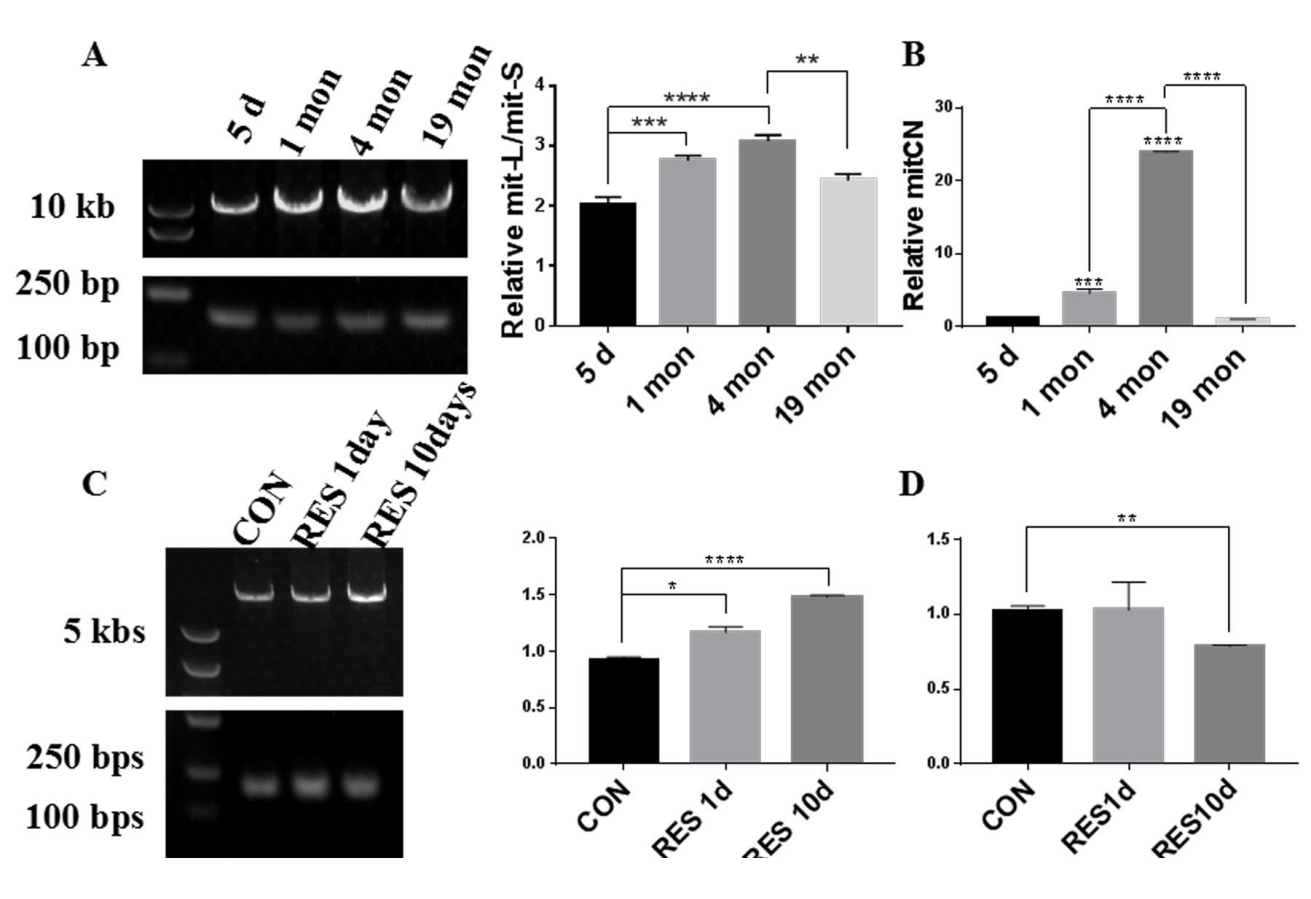
**Zebrafish retinal mtDNA quality at different ages and after resveratrol treatment.** (**A, C**) The bands in the left lane are DNA size markers. The top slice shows the long mitochondrial fragments (mit-L) while the bottom slice shows the short mitochondrial fragments (mit-S). The graphs show the ratio of mit-L/mit-S by densitometry analysis of each band (mean ± SEM, *P<0.05, **P<0.01, ***P<0.001, ****P<0.0001, n=3) (**B**) Comparison of mitochondrial copy number (mitCN) in 5 days, 1 month, 4 months, and 19 months old zebrafish retinas (mean ± SEM, ***P<0.001, ****P<0.0001 , n=3.) (**D**) Comparison of mitCN in zebrafish retinas after treatment with 20mg/L resveratrol for 1 or 10 days (mean ± SEM, **P<0.01 compared to control, n=3.). CON, control. RES1d, resveratrol treated for 10 days; RES10d, resveratrol treated for 10 days.

To investigate the mitochondrial quality in aging zebrafish retinas, we first analyzed the ratio of mitochondrial long chain (mit-L) to mitochondrial short chain (mit-S) DNA which reflects the mitochondrial integrity and the ability to repair mitochondrial damage. Zebrafish retinas exhibited varying levels of mitochondrial integrity at different ages. While mitochondrial integrity was higher at 1 and 4 months compared to 5 days old, at 19 months old the mitochondrial integrity had returned to the level of the 5-day old retinas (Figure 1A). mtDNA copy number (mtCN) is proportional to mitochondrial mass, and we observed that at 4 months old the mtDNA copy number of retinas was substantially increased compared to 5-day and 1-month old retinas, however at 19 months it had also returned to the level of 5-day old retinas (Figure 1B). Resveratrol treatment of young zebrafish significantly increased the mitochondrial integrity after 1 day and 10 days treatment (Figure 1C). Interestingly, resveratrol did not alter the mtCN after 1 day of treatment but slightly decreased it after 10 days treatment (Figure 1D).

### Mitochondrial fusion/fission were unbalanced in aging zebrafish retina and improved by resveratrol treatment

**Figure 2:**
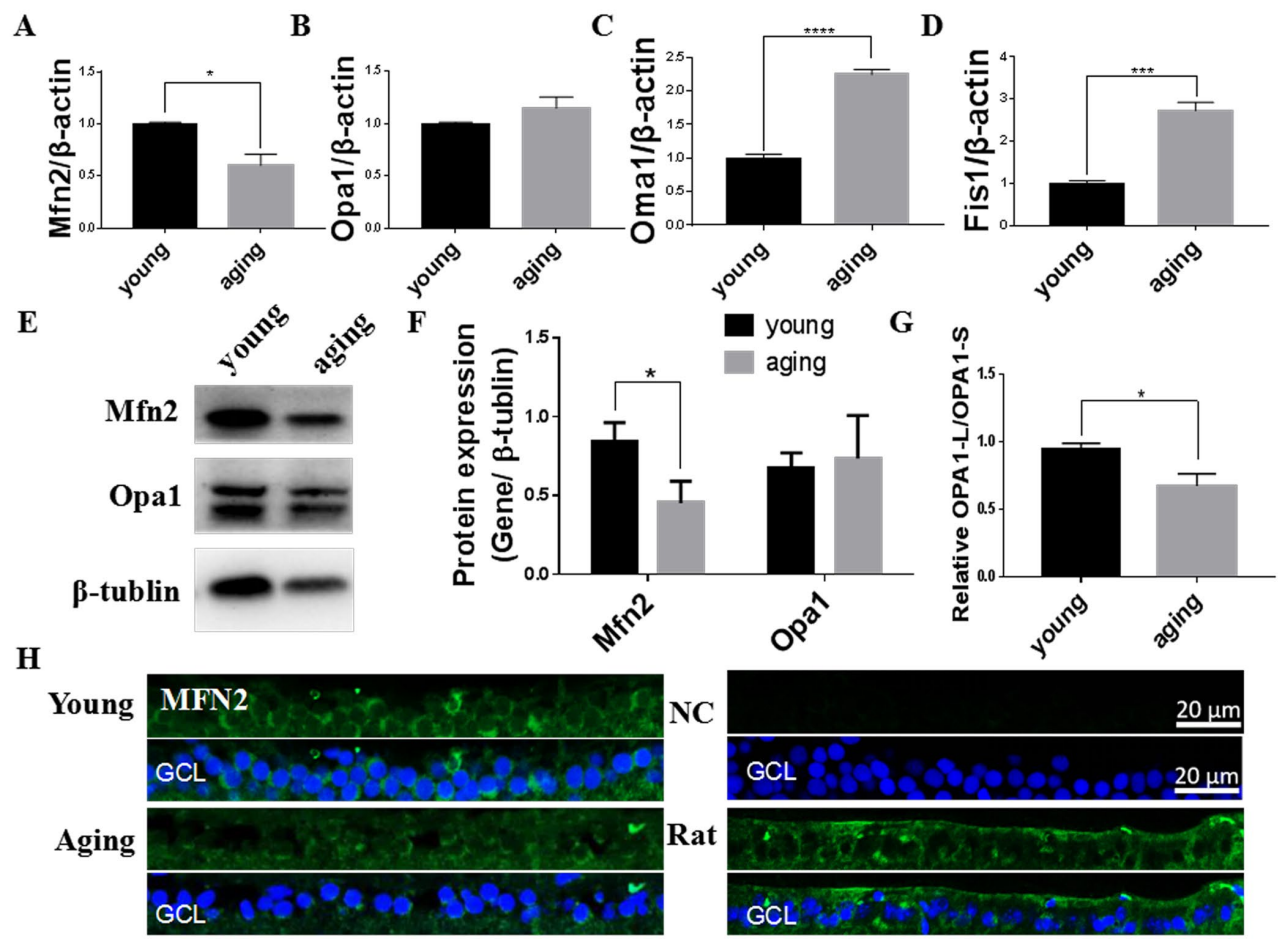
**Mitochondrial fusion/fission imbalanced in aging zebrafish retinas.** (**A-D**) Mfn2, Opa1, Oma1, and Fis1 expression in young and aging zebrafish retinas. Graphs represent **(A)** Mfn2, (**B**) Opa1, (**C**) Oma1, and (**D**) Fis1 gene expression by quantitative real-time PCR (mean ± SEM, *P<0.05, ***P<0.001, ****P<0.0001, n=3. (**E**) Representative western blot showing the protein expression levels of Mfn2 and Opa1 in young and aging zebrafish retinas. (**F**) The graph depicts the densitometric mean and SEM normalized to the corresponding level of the loading control protein beta-tubulin (*P<0.05, n=3). (**G**) The ratio of Opa1-L/Opa1-S, as determined by densitometry of western blots as in E (*P<0.05, n=3**. (H**) Immunofluorescence localization and relative expression of Mfn2 in the RGC layer of young and aging zebrafish retina cross-sections. All photographs were taken at 40x magnification except rat sections were at 20x magnification. Young, 4-6 months old zebrafish; aging, 19-23 months old zebrafish; NC, negative control (no primary antibody); Rat, rat retina positive control; Opa1-L, Opa1 long segment; Opa1-S, Opa1 short segment; GCL, ganglion cell layer.

**Figure 3:**
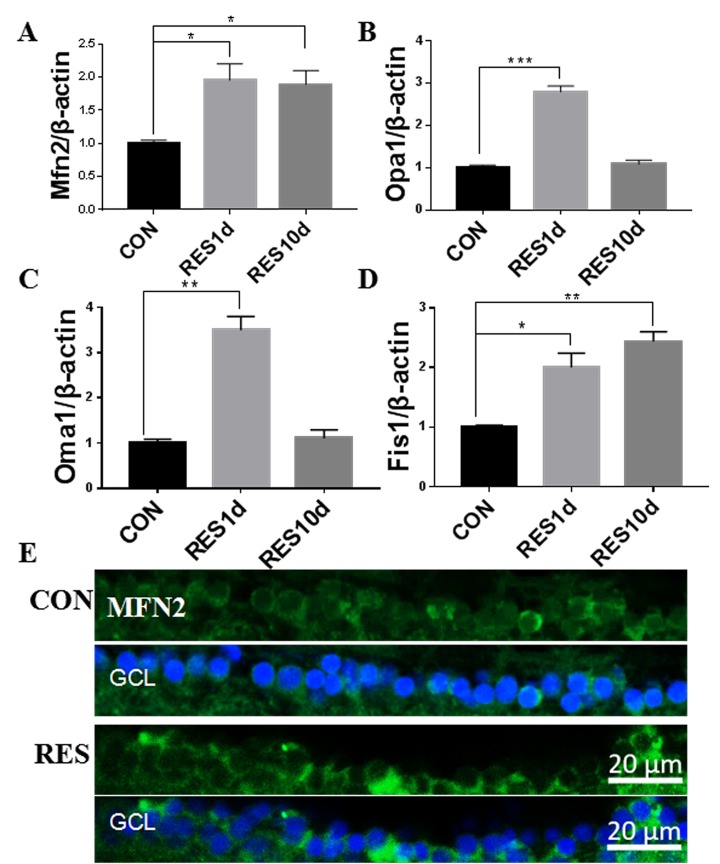
**Resveratrol treatment increased mitochondrial fusion/fission expression in young zebrafish retina.** (**A-D**) Mfn2, Opa1, Oma1, and Fis1 gene expression after resveratrol treatment for 1 or 10 days as determined by quantitative real-time PCR (mean ± SEM, *P<0.05, **P<0.01, ***P<0.001 compared to control (CON), n=3). (**E**) Immunofluorescence localization and relative expression of Mfn2 in the RGC layer of adult zebrafish retina cross-sections after 10 days resveratrol treatment. All photographs were taken at 40x magnification. CON, control; RES1d, resveratrol treated for 1 days; RES/RES10d, resveratrol treated for 10 days; GCL, ganglion cell layer.

Mitochondrial dynamics is vital for maintaining mitochondrial quality, with fusion used to increase mitochondrial integrity and fission to increase mitCN [10]. We found Mfn2, one of the primary mitochondrial fusion regulators, was decreased in aging retinas compared to young retinas at both the mRNA and protein level (Figure 2A, E, F). This was particularly pronounced in the cytoplasm of the retinal ganglia cell layer (GCL) [41] (Figure 3H). Although the level of total Opa1, the other mitochondrial fusion regulator, was not significantly altered at the gene or protein level between young and aging retinas (Figure 2B, E, F), the ratio of long Opa1-L to short Opa1-S was decreased in the aging retinas (Figure 2G). Moreover, expression of Oma1, the metalloprotease that cleaves functional Opa1-L into the inactive Opa1-S, was increased in aging retinas (Figure 2C). Expression of the mitochondrial fission regulator gene Fis1 was increased in aging retinas versus young retinas (Figure 2D). Together, this suggests that mitochondrial fission is promoted in aging retinas through decreased Mfn2 expression, proteolytic inactivation of Opa1 by Oma1, and increased Fis1 expression.

Resveratrol increased Mfn2 gene expression after both 1- and 10-days treatment in the young zebrafish. Opa1 and Oma1 gene expression increased after 1-day treatment but returned to their original levels after 10 days treatment, while Fis1 gene expression was steadily increased after 1- and 10-days treatment with resveratrol **(**Figure 3A-D). Increased cytoplasmic levels of Mfn2 in the RGC layer is also apparent upon resveratrol treatment (Figure 3E).

### Mitophagy was decreased in the aging zebrafish retina and increased by resveratrol treatment

**Figure 4:**
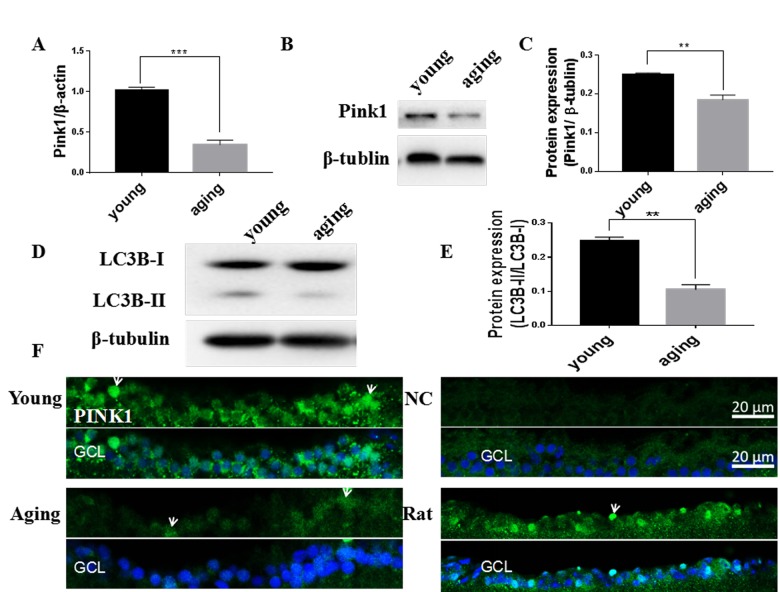
**Decreased mitophagy and autophagy in aging zebrafish retinas.** (**A**) Pink1 gene expression in young and aging zebrafish retinas as measured by quantitative real-time PCR (mean ± SEM, ***P<0.001, n=3). (**B**) Representative western blot showing the protein expression level of Pink1 in young and aging zebrafish retinas. (**C**) Graph of the densitometric mean and SEM normalized to the corresponding level of the loading control protein beta-tubulin (**P<0.01, n=3). (**D**) Representative western blot showing the protein expression levels of LC3B-II and LC3B-I in young and aging zebrafish retina. (**E**) Graph of the ratio of LC3B-II to LC3B-I protein levels as determined by densitometry (**P<0.01, n=3). (**F**) Immunofluorescence analysis of Pink1 in the RGC layer of young and aging zebrafish retinas. Retina cross sections prepared from zebrafish eyes were immunostained with Pink1 antibody. All photographs were taken at 40x magnification except the rat sections that were at 20x. Young, 4-6 months old; aging, 19-23 months old; NC, negative control (without primary antibody); Rat, positive control; GCL, ganglion cell layer.

Mitophagy can both degrade damaged mitochondria and control the overall mitochondrial content of the cell [19]. Compared to young retinas, we observed that mRNA and protein levels of Pink1, a central regulator of mitophagy, decreased in aging zebrafish retinas (Figure 4A-C). The decline in Pink1 expression was also observed in the nuclei and cytoplasm of the RGC layer of aging retinas [42] (Figure 4F). Microtubule-associated protein 1B light chain 3B (LC3B) is a marker of autophagy, and the ratio of LC3B short segment (LC3B-II) to LC3B long segment (LC3B-I) is representative of the level of autophagy [43]. Consistent with decreased mitophagy, there was also a significant decrease in the LC3B-II/LC3B-I ratio in aging versus young retinas, suggesting a decrease in overall autophagy levels (Figure 4D, E).

**Figure 5:**
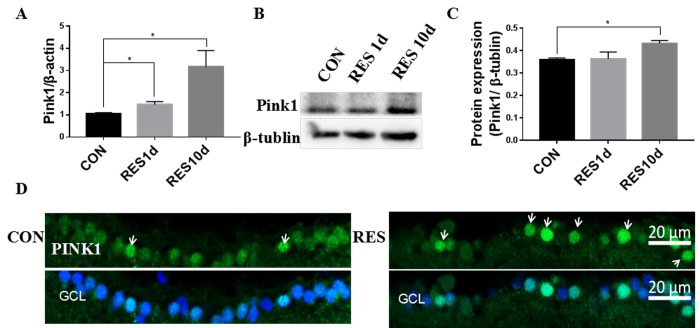
**Resveratrol treatment increased mitophagy in young zebrafish retina.** (**A**) Pink1 gene expression in adult zebrafish retina after treatment with resveratrol for 1 and 10 days as measured by quantitative real-time PCR (mean ± SEM, *P<0.05 compared to control (CON), n=3). (**B**) Representative western blot showing the protein expression of Pink1 in adult zebrafish retina after treatment with resveratrol for 1 and 10 days. (**C**) Graph of the densitometric mean and SEM normalized to the corresponding level of the loading control protein beta-tubulin from western blots as in B (*P<0.05 compared to control (CON), n=3). (**D**) Immunofluorescence localization and relative expression of Pink1 in the RGC layer of adult zebrafish retinas after treatment with resveratrol for 10 days. Retina cross sections prepared from zebrafish eyes were immunostained with Pink1 antibody. All photographs were taken at 40x magnification. CON, control; RES1d, resveratrol treated for 1 days; RES/RES10d, resveratrol treated for 10 days; GCL, ganglion cell layer.

Young zebrafish treated with resveratrol for 1 or 10 days had increased Pink1 mRNA and protein levels (Figure 5A-C), which was also observable in the nuclei and cytoplasm of the RGC layer (Figure 5D).

### The Akt/mTOR pathway was up-regulated in aging zebrafish retinas and down-regulated by resveratrol treatment

**Figure 6:**
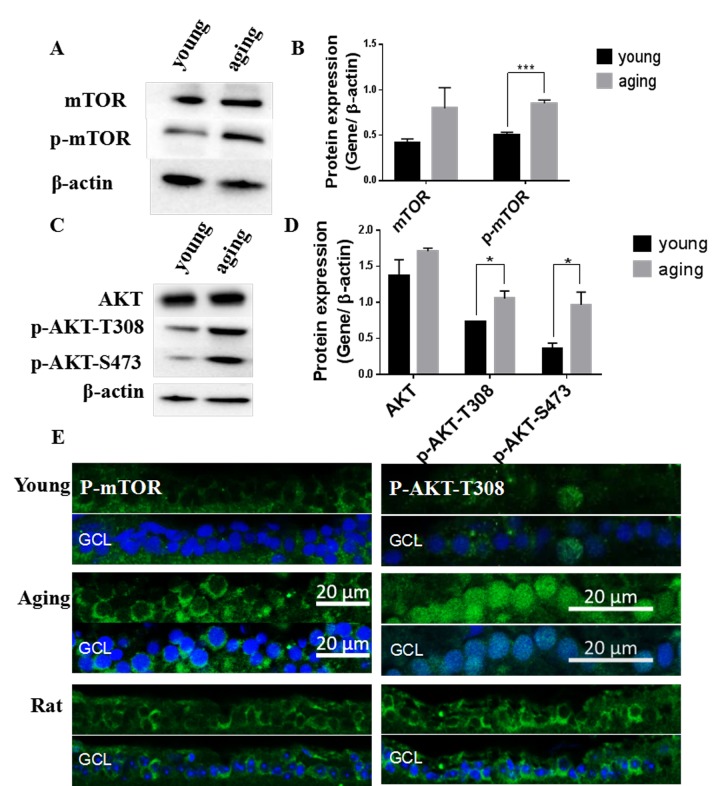
**Activation of the Akt/mTOR pathway in aging zebrafish retinas.** (**A**) Representative western blot showing the protein level of mTOR and p-mTOR in young and aging zebrafish retinas. (**B**) Densitometric mean and SEM normalized to the corresponding level of the loading control protein beta-actin (***P<0.001, n=3. (**C**) Representative western blot showing the protein expression level of Akt and p-Akt-T308 and p-Akt-S473 in young and aging zebrafish retinas. (**D**) Densitometric mean and SEM normalized to the corresponding level of the loading control protein beta-actin (*P<0.05, n=3. (**E**) Photographs of fluorescent immunostained retina cross-sections showing the localization and relative levels of p-mTOR and p-Akt-T308 in the RGC layers of young and aging zebrafish retinas. All photographs were taken at 40x magnification except rat sections were at 20x magnification and p-Akt-T308 at 60x magnification. Young, 4-6 months old zebrafish; Aging, 19-23-month-old zebrafish; Rat, positive control; GCL, ganglion cell layer.

Both autophagy and mitophagy are regulated by the protein kinase B (Akt)/mTOR pathway [9,16]. In this study, we observed that the total protein level of mTOR as well as mTOR phosphorylation (p-mTOR) were up-regulated in aging retinas compared to young retinas (Figure 6A-B). The upstream activator of mTOR, Akt, also exhibited increased phosphorylation at threonine 308 (p-Akt-T308) and serine 473 (p-Akt-S473) (Figure 6C-D). The increased p-mTOR and p-Akt-T308 in aging retinas was also observed by immunostaining the RGC layer [44,45] (Figure 6E). The elevated p-mTOR signal was primarily localized in the cytoplasm whereas the elevated p-Akt-T308 was largely located in the nuclei of aging zebrafish retinas (Figure 6E). In contrast, rat retinas exhibited p-Akt-T308 only in the cytoplasm, suggesting that there may be species-specific differences (Figure 6E).

**Figure 7:**
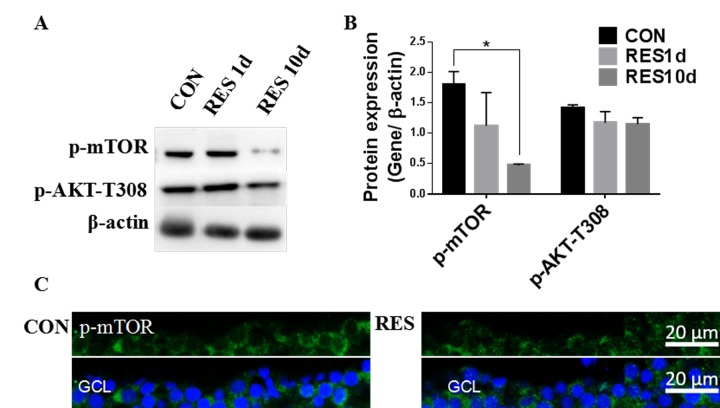
**Resveratrol treatment suppressed the Akt/mTOR pathway in young zebrafish retinas.** (**A**) Representative western blot showing the levels of p-mTOR and p-Akt-T308 in adult zebrafish retinas after being treated with resveratrol for 1 and 10 days. (**B**) Densitometric mean and SEM of p-mTOR and p-Akt-T308 normalized to the corresponding level of the loading control protein beta-actin (*P<0.05 compared to control (CON), n=3). (**C**) Photographs of fluorescence immunostained retina cross-sections showing the localization and relative levels of p-mTOR in the RGC layers of adult zebrafish retinas with or without resveratrol treatment for 10 days. All photographs were taken at 40x magnification. CON, control; RES1d, resveratrol treated for 1 days; RES/RES10d, resveratrol treated for 10 days; GCL, ganglion cell layer.

Resveratrol treatment led to a significant decrease in p-mTOR, and had a small but insignificant effect on p-AKT-T308 in young zebrafish retinas (Figure 7 A-B). Immunofluorescence showed that p-mTOR was decreased in the RGC layers of resveratrol-treated young zebrafish retinas (Figure 7C).

### Resveratrol treatment activated Mfn2, Fis1, Pink1, and suppressed p-mTOR in aging zebrafish retina

**Figure 8:**
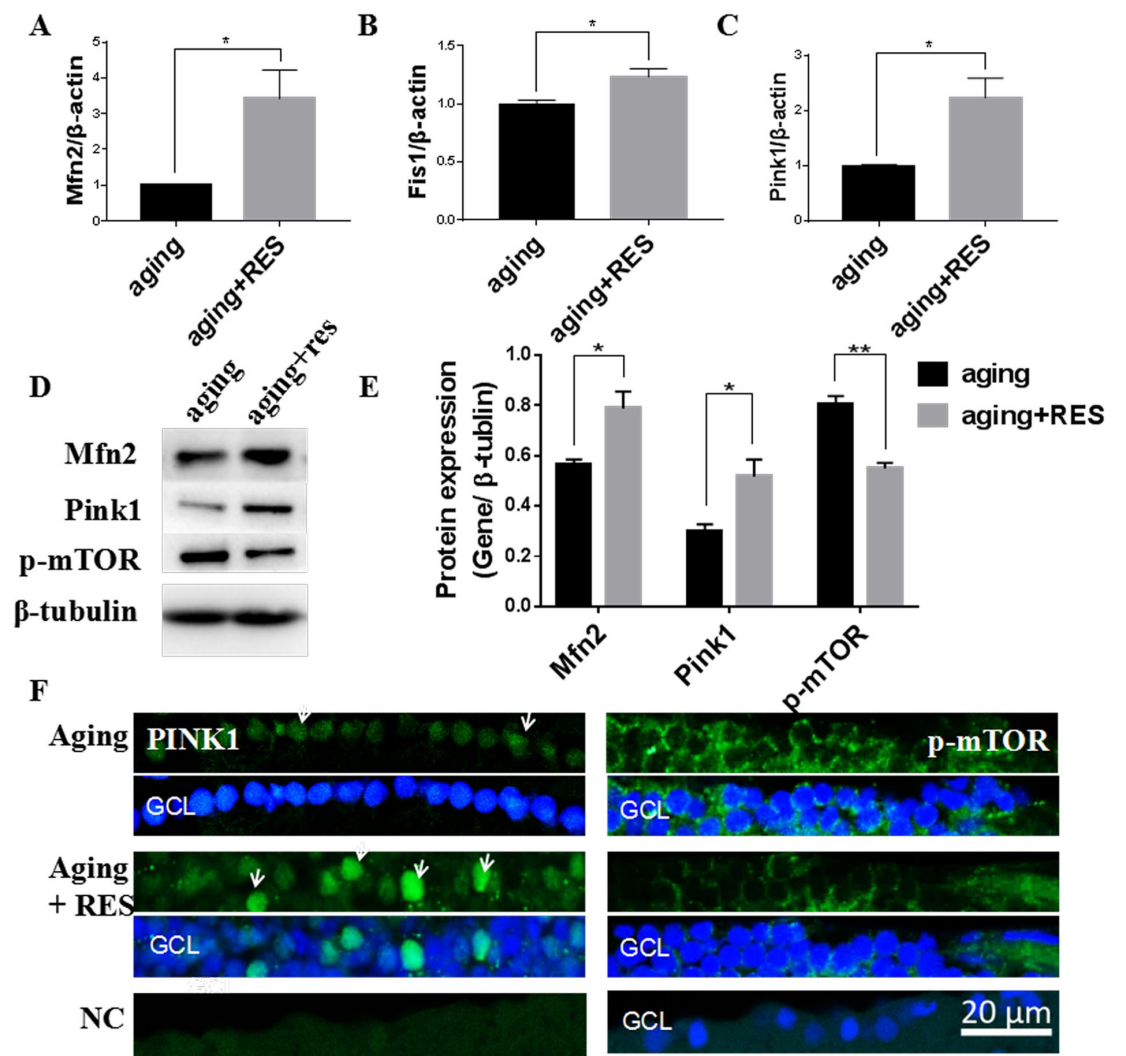
**Resveratrol treatment activated Mfn2, Fis1, Pink1, and suppressed p-mTOR in aging zebrafish retina.** (**A-C**) Mfn2, Fis1, Pink1 expression in aging and resveratrol-treated aging zebrafish retina. Graphs represent **(A)**Mfn2 (**B**) Fis1(**C**) Pink1 gene expression by quantitative real-time PCR (mean ± SEM, *P<0.05, n=3). (**D**)Representative western blot showing the protein expression levels of Mfn2,Pink1,p-mTOR in aging and resveratrol-treated aging zebrafish retina. (**E)**The graph showing the densitometric mean and SEM normalized to the corresponding level of the loading control protein beta-tubulin (*P<0.05, **P<0.01, n=3). (**F**) Immunolocalization and relative quantitative expression of Pink1 and p-mTOR on RGC in aging and resveratrol-treated aging zebrafish retina cross-sections. Retina cross sections prepared from zebrafish eyes were immunostained with Mfn2 or p-mTOR antibody. Results present aging, resveratrol-treated aging, and negative control (without primary antibody). All photographs were taken at 40 times. aging, 19-23 months zebrafish; aging + RES, resveratrol-treated aging zebrafish for 10 days; NC, negative control; GCL, ganglion cell layer.

Similar to the young zebrafish, we observed that resveratrol also increased Mfn2 and Fis1 and Pink1 expression in aging zebrafish retinas (Figure 8). And Importantly, p-mTOR was also decreased in the aging zebrafish retinas by resveratrol (Figure 8D-E, F).

### Expression of antioxidant defense enzymes was suppressed in aging zebrafish retinas and increased by resveratrol treatment

**Figure 9:**
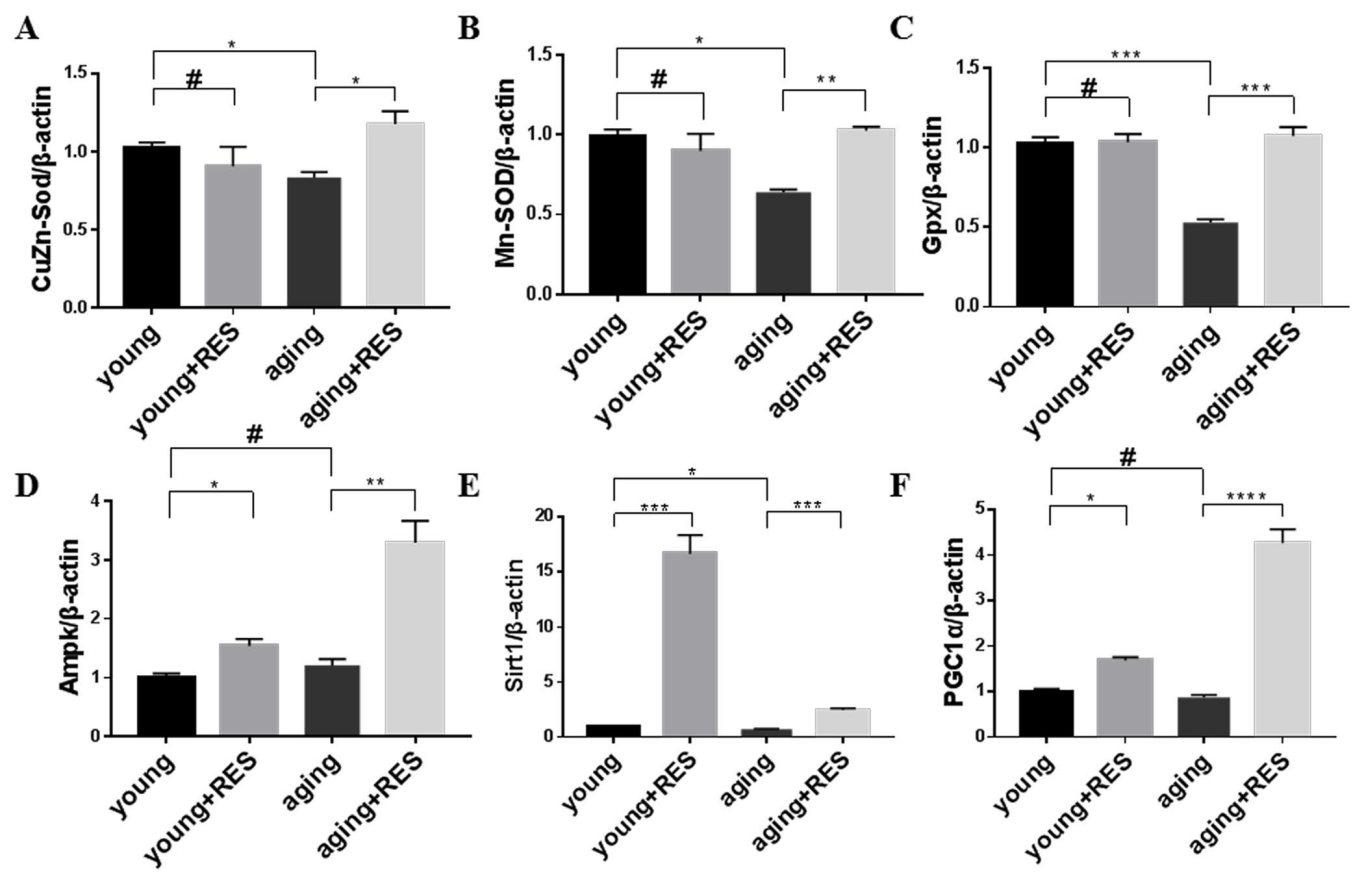
**Resveratrol treatment activated the antioxidant system and Ampk/Sirt1/Pgc1α pathway in aging zebrafish retina.** (**A-F**) Cu/Zn-Sod, Mn-Sod, Gpx, Ampk, Sirt1, and Pgc1α expression in young, resveratrol-treated young, aging, and resveratrol-treated aging zebrafish retinas. Graphs represent (**A**) Cu/Zn-Sod, (**B**) Mn-Sod, (**C**) Gpx, (**D**) Ampk, (**E**) Sirt1, and (**F**) Pgc1α gene expression by quantitative real-time PCR (mean ± SEM, *P<0.05 **P<0.01 ***P<0.001 ****P<0.0001,#P means no statistical significance, n=3). young, 4-6 month old zebrafish; young + RES, young zebrafish after resveratrol treatment for 10 days; aging, 19-23 month old zebrafish; aging + RES, aging zebrafish after resveratrol treatment for 10 days.

The cellular ROS detoxification system includes the Superoxide dismutase (Sod) and Glutathione peroxidase (Gpx) proteins [8]. Consistent with increased oxidative damage in aged tissues, we observed decreased expression of Cu/Zn-Sod, Mn-Sod, and Gpx in aging zebrafish retinas (Figure 9A-C). Although resveratrol had no significant effect on Cu/Zn-Sod, Mn-Sod, or Gpx expression in young zebrafish, it did increase expression of all three in aging zebrafish retinas (Figure 9A-C).

### Resveratrol activated Ampk/Sirt1/Pgc1α in zebrafish retinas

The Ampk/Sirt1/Pgc1α pathway is crucial for regulating mitochondrial biogenesis and oxidative metabolism [9]. We observed that resveratrol increased Ampk, Sirt1, and Pgc1α expression in both young and aging zebrafish retinas (Figure 9D-F). For Sirt1, young zebrafish had a greater response to resveratrol, while for Ampk and Pgc1α the aging zebrafish had a higher increase due to resveratrol (Figure 9D-F).

### Inflammation elevated in aging zebrafish retina and not regulated by resveratrol treatment

**Figure 10:**
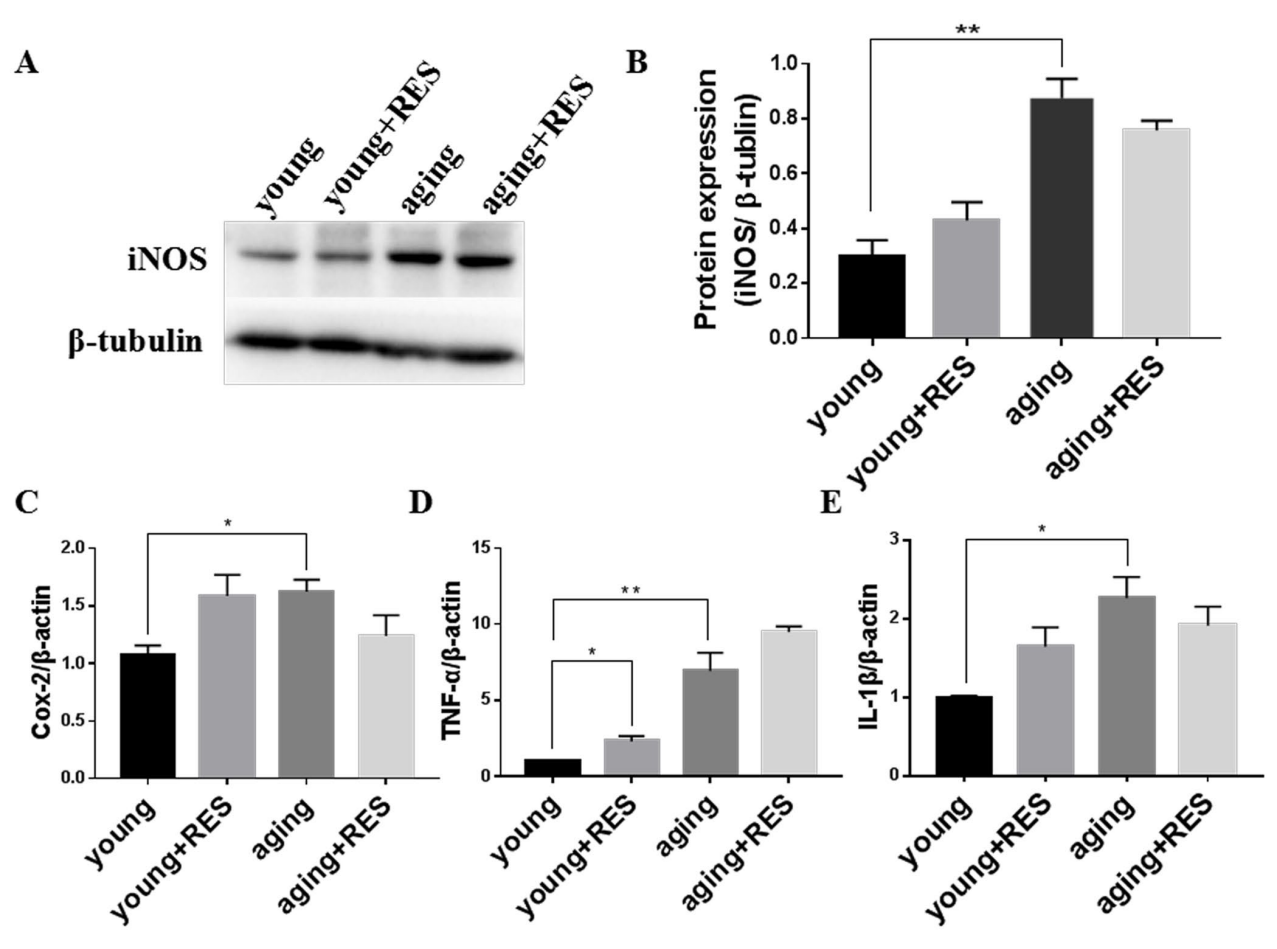
**Inflammation elevated in aging zebrafish retina and not regulated by resveratrol treatment** (**A**) Representative western blot showing the protein expression level of iNOS in young, resveratrol-treated young, aging, and resveratrol-treated aging zebrafish retinas. (**B**) The densitometric mean and SEM of bands from **A** normalized to the corresponding level of the loading control protein beta-tubulin (**P<0.01, n=3). (**C-E**) Cox-2, TNF-α, and IL-1β expression in young, resveratrol-treated young, aging, and resveratrol-treated aging zebrafish retinas. Graphs represent (**C**) Cox-2, (**D**) TNF-α, and (**E**) IL-1β gene expression by quantitative real-time PCR (mean ± SEM, *P<0.05 **P<0.01, n=3). young, 4-6 month old zebrafish; young + RES, young zebrafish after resveratrol treatment for 10 day; aging, 19-23 month old zebrafish; aging + RES, aging zebrafish after resveratrol treatment for 10 days.

We found in aging zebrafish retina, expression of inflammation-related protein iNOS were increased, and inflammation-related genes Cox-2, TNF-α and IL-1βwere also increased (Figure 10) [46]. And resveartrol had no significant effect in young and aging zebrafish.

## DISCUSSION

Mitochondrial dysfunction has been implicated as a leading cause of aging-related oculopathy, a very common ailment worldwide that can lead to visual impairment and blindness [7,8]. In the present study, we used zebrafish as a model system to study aging-related alterations in retinas including mtDNA integrity, mtDNA copy number, mitochondrial fusion/fission regulators, mitophagy and autophagy, Akt/mTOR, Ampk/Sirt1/Pgc1α and inflammation pathway activity. We also explored the ability of the anti-aging natural product resveratrol to modulate age-related alterations to the zebrafish retina.

We first explored mitochondrial quality and found that mtDNA integrity was lower in aging zebrafish retinas than in retinas of young zebrafish, suggesting that the mtDNA has suffered significant damage during aging [12,47]. Accumulation of ROS with age will harm mtDNA in particular, since mtDNA is more sensitive to oxidative damage due to its proximity to ROS production in the mitochondrial membrane. Moreover, a weaker antioxidant defense system in aging retinas further increases the mutation rate. The increased mutation load can then lead to a loss of mtDNA integrity [10,48,49]. Loss of mitochondrial content during aging has also been reported, and mitochondrial mass and mitochondrial copy number (mitCN) are decreased in neurodegeneration [31,50]. The mitCN was sharply decreased in aging compared to young retinas, indicating that the function of retinal mitochondria and their ability to efficiently produce energy are compromised during aging. Retinal cells and other neurons are particularly high energy-demanding tissues, so mitochondrial dysfunction has a higher impact on them compared to other tissues [9,51].

We observed that the mtDNA integrity of young zebrafish retinas was increased after 1- or 10-days treatment with resveratrol, whereas the mitCN had a slight decrease after 10 days treatment but was not altered after only 1 day of treatment. One previous study found that resveratrol for one day can increase mtDNA integrity (the ratio of mit-L to mit-S) in adult zebrafish retinas but did not change the mitCN [30]. Another report indicated that resveratrol can decrease mitCN in mouse oocytes [52]. Our current results may be explained by an increase in mitophagy between 1 and 10 days of resveratrol treatment, which would increase the mitochondrial integrity by increasing the turnover of damaged mtDNA and thus decreasing the total mass of mitochondria.

Rather than existing as separate, individual units, a cell’s mitochondria together make up a dynamic network of organelles in a constant state of flux between fusion and fission to regulate mitochondrial quality, maintain critical cellular functions, and ensure an adequate supply of energy. A healthy mitochondrial fusion/fission flux should balance the elimination of damaged mitochondria with the rate of new mitochondrial production to maintain mitochondrial homeostasis [10,44]. Mfn2 and Opa1 are vital regulators of fusion and are located at the outer- and inner-mitochondrial membrane, respectively, while Fis1 is a vital regulator of fission and is located at the outer-mitochondrial membrane [10,31,53]. Opa1 is a multi-domain protein that is cleaved by Oma1 from a long isoform (Opa1-L) into a short isoform (Opa1-S). Opa1-L increases mitochondrial fusion, while Opa1-S increases fission, and therefore reduced Oma1 expression is believed to prolong lifespan because more Opa1-L will be present to prevent accumulation of damaged mitochondria. On the other hand, more Opa1-S may allow accumulation of dysfunctional mitochondria [54–56]. Fis1, the mitochondrial fission regulator, is also associated with age-related neurodegeneration, and has been observed to be increased in AD and HD patient tissues [52,57]. In our study, we found that Mfn2 gene and protein expression was decreased, Opa1 gene and protein expression was unchanged, and Fis1 gene expression was increased in aging zebrafish retinas. These changes are consistent with previous reports and further support the notion that regulators of mitochondrial dynamics are mediators of aging in retinas as well as other neurons implicated in neurodegenerative diseases [5,53,58]. Moreover, our study was the first to show that Oma1 gene expression and the ratio of Opa1-L/Opa1-S was decreased during aging.

We postulated that mitochondria in aging zebrafish retinas are tipped to increased fragmentation, as the fusion activators are suppressed and fission activators are increased. Fragmentation is a compensatory means of adaptation to a low nutrition environment caused by ROS accumulation and loss of functional mitochondria. However, in the long term the vicious circle of increased fission and decreased fusion aggravates the aging process and likely plays a causative role in neurodegenerative diseases [5,11,21,44].

Many studies have provided evidence that resveratrol can improve mitochondrial function, particularly in old age, but the mechanisms remain unclear [31]. In the current study, we found that Mfn2, Opa1, Oma1, and Fis1 expression were all increased after 1 day of resveratrol treatment, but Opa1 and Oma1 levels returned to the level of control after 10 days treatment while Mfn2 and Fis1 remained elevated. Although resveratrol may act acutely on some genes that are nevertheless important for its beneficial effects, we postulated that the protective effects of resveratrol on mitochondrial dynamics may be primarily mediated by Mfn2 and Fis1 [30]. Kaige Peng et al. found that resveratrol can improve mitochondrial fusion-fission dynamics by promoting Mfn2, Opa1, and Fis1 expression to protect against the rotenone-induced neurotoxicity that would otherwise lead to mitochondrial homeostasis disorder, cellular damage, and dopaminergic neuron degeneration [31]. Although some suggest that inhibiting fission may be a beneficial target for neurodegenerative diseases, we believe that activating both fusion and fission together would be a better option [52]. Resveratrol, for example, appears to both promote fusion to dilute mitochondrial damage while also promoting fission to provide more energy generation and permit the elimination of unhealthy mitochondria through fission and mitophagy [10,23].

As the name suggests, mitophagy is the specialized autophagic process that digests unhealthy or excess mitochondria [59]. The relationship between autophagy and mitophagy is regulated by the p62 protein, which in vertebrates regulates autophagy via interacting with LC3 and mitophagy by controlling the Pink1/Parkin pathway [60]. We observed that the ratio of LC3B-II/LC3B-I protein, a marker of autophagy activity, was decreased in aging zebrafish retinas. Gene and protein expression of Pink1, a vital regulator of mitophagy, was also decreased in aging zebrafish retinas. Dysfunction of mitophagy and autophagy is implicated in neurodegenerative diseases including HD, AD, and Parkinson’s disease (PD), where dysfunction of mitophagy and autophagy can lead to mitochondrial dysfunction and cell death [60]. The study by Wu et al. indicate that mitophagy and autophagy down-regulate mitochondrial ROS production through NLRP3 inflammasome activation [61], while a study by Lutz found an increase in mitochondrial fragmentation after loss of Pink1/Parkin function [62]. In the present study, we found that resveratrol can promote Pink1 gene and protein expression after 1- and 10-days treatment. Several other reports also showed that resveratrol can induce autophagy in neurons and promote Pink1/Parkin expression [21,34]. Over-expression of mitophagy activators can prolong drosophila and mouse lifespan, which occurred due to the activation of both fusion/fission and mitophagy [17,63,64].

Accumulation of mitochondrial damage will lead to aging, so it is of interest why cells would down-regulate protective mechanisms such as mitochondrial fusion/fission and mitophagy/autophagy who maintain mitochondrial activity during aging. Many studies have pointed to mTOR as a culprit in aging, showing that elevated mTOR activity can affect mitochondrial function and inhibit mitophagy/autophagy, thus contributing to the aging process [16,65]. Activated mTOR inhibits autophagy (including mitophagy), whereas inhibition of the Akt/mTOR pathway induces autophagy and increases the lifespan of many species [9,43,66]. Moreover, downregulation of mTOR has been shown to increase longevity by maintaining mitochondrial function and appropriate energy consumption [63,65,67]. Increased mTOR activity is observed in aging tissues, and many believe that the PI3K/PTEN/Akt/mTOR pathway is one of the most important regulators of aging [68].

We found both increased expression and phosphorylation (indicating activation) of mTOR and Akt in aging zebrafish retinas. Moreover, resveratrol caused a significant decrease in p-mTOR after 10 days treatment, while p-Akt-T308 had no significant change. These results are consistent with those of Jun Wu et al. who found that resveratrol could promote mitophagy/autophagy in HMrSV5 cells by suppressing p-mTOR, and who also demonstrated that LC3-II accumulated with mTOR knockout [61].

Regulation of mitochondrial function by mTOR is not solely through its regulation of mitophagy/autophagy [16]. Several reports suggest that mTOR can activate HIF-1, PGC1α, YY-1, and AMPK to regulate mitochondrial oxygen consumption, mitochondrial biogenesis, and mitochondrial metabolism [67,69,70]. Results from Masahiro Morita et al. suggest that the nutrient-sensing ability of mTOR promotes mitochondrial fission, and suppressing mTOR can elevate mitochondrial fusion to protect cells from mitochondrial degeneration [71]. It is therefore believed that mTOR inhibition should be targeted to protect neurons against age-related neurodegenerative diseases [72]. The current gold standard pharmacological inhibitor of mTOR is rapamycin, an extremely effective inhibitor of mTOR Complex 1, but it can cause many side effects including hyperglycemia, hyperlipidemia, anemia, eye diseases, and some testicle diseases [73]. Therefore, searching for other safer mTOR inhibitors that can improve mitochondrial function is required.

Given the relationships between aging, mitochondrial function, inflammation, and ROS, we explored what effects resveratrol has on the cellular ROS detoxification system. Our prior study showed that I/R-induced inflammation can promote ROS accumulation and subsequent cellular damage in the rat retina leading to cell death, and that resveratrol could attenuate this inflammation and protect retinas [36]. However, in the current study we found that although expression of inflammation-related proteins was indeed increased in aging zebrafish retinas, resveratrol had no significant effect on their expression in young or aging zebrafish (Figure 10). And we found that in aging zebrafish retina, the antioxidant system such as CuZn-Sod, Mn-Sod and Gpx were decreased, which indicated the ability of cell to clean oxidative products was down-regulated, and the ROS level was increased in aging [9]. The result that resveratrol can activate oxidant system in aging but had no effect in young zebrafish retina illustrated resveratrol can maintian antioxidant system at a suitable level to improve mitochondrial quality and energy metabolism.

The Ampk/Sirt1/Pgc1α axis is considered to be a critical sensor of metabolic demand and regulator of energy production and consumption [9,74]. By increasing levels of Ampk, Sirt1, and Pgc1α, resveratrol can enhance mitochondrial biogenesis, oxidative metabolism, and cellular ROS defense enzymes to cope with the stress of aging in tissues such as the retina with a high metabolic demand. Increasing Ampk/Sirt1 activity can also inhibit mTOR downstream to promote mitophagy. Some reports also suggest that Sirt1/Pgc1α activation can help prevent mitochondrial fragmentation to alleviate mitochondrial dysfunction [75]. Therefore, activation of the Ampk/Sirt1/Pgc1α pathway by resveratrol is likely crucial for its ability to maintain healthy mitochondrial dynamics and regulate ROS levels for healthy aging.

**Figure 11:**
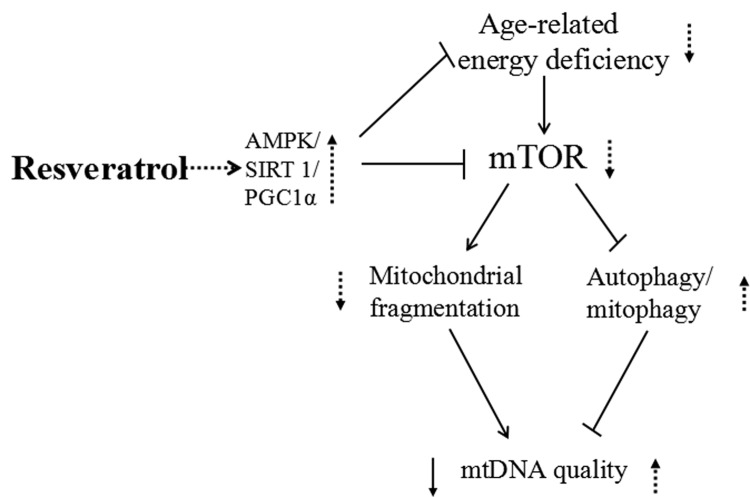
**Schematic representation of resveratrol induced activation of AMPK/ SIRT1/ PGC1α and inhibition of mTOR, leading to increased mitochondrial quality.** Resveratrol treatment enhances AMPK/SIRT1/PGC1α expression and decreases mTOR signaling, which leads to an increase in mtDNA quality and enhanced energy metabolism at least partly through decreased mitochondrial fragmentation and increased autophagy/mitophagy.

Zebrafish has been utilized to study many age-related eye diseases including cataracts, glaucoma, and age-related macular degeneration (AMD). For example, NMDA treatment damages retinal neurons and was used to establish a zebrafish glaucoma model, while the gnn mutant or vascular endothelial growth factor (VEGF) treatment lead to AMD characteristics similar to those seen in human AMD [30,37,76,77].We postulate that in aging zebrafish retinas the high demand for nutrition and energy will activate mTOR, which promotes mitochondrial fission to increase ATP production and suppresses fusion to compensate for the decreasing mitochondrial function. Increased oxygen consumption and inflammation leads to accumulation of excess ROS and an increase in oxidative to damage mtDNA. Mitophagy/autophagy are suppressed by mTOR, causing a buildup of unhealthy mitochondria that cannot be eliminated and this mitochondrial fragmentation can promote aging. Many studies have shown that resveratrol has anti-aging and neuroprotective properties, although the mechanisms are not completely understood [31]. We explored the effects of resveratrol on mtDNA intergrity and copy number, mitochondrial fusion/fission, mitophage and anti-ROS regulators, Akt/mTOR and Ampk/Sirt1/Pgc1α pathway, and found that it was able to alleviate or diminish many of the detrimental changes that occur during aging. Further studies are needed to analyze whether resveratrol can protect against ROS accumulation and whether its beneficial molecular effects can translate to protection of the retina against age-related oculopathy (Figure 11).

## Conclusions

Maintaining mitochondrial health is crucial to prevent age-related neurodegenerative diseases, including oculopathy. We utilized the zebrafish retina as a model for age-related oculopathy and observed decreased mtDNA integrity, dysfunctional mitochondrial fission-fusion dynamics, decreased expression of antioxidant defense enzymes, and increased activity of the Akt/mTOR pathway in the aging retina. Consistent with its anti-aging effects in other species and model systems, resveratrol treatment helped alleviate most of the age-related changes observed in the zebrafish retina, suggesting its potential for the prevention of aging-induced oculopathy in other species including humans. This study indicates that further testing of resveratrol for oculopathy is warranted, and helps establish the zebrafish retina as a viable model of age-related oculopathy for further studies on the molecular mechanisms and for novel drug screening.

## MATERIALS AND METHODS

### Animals

Male and female 4-6 months old ( young/control) and 19-23 months old (aged) wild-type zebrafish of AB strain were obtained from the China Zebrafish Resource Center (CZRC, Wuhan, China) [40], and 5 days and 1 month old zebrafishes were offspring of the former. All fish were maintained in a 25 L aquarium at 28°C under 14-hour/10-hour light/dark cycles and were fed with brine shrimp twice a day. The maintenance and experimental manipulations of zebrafish were approved by the ethical review committee of Nanchang University (Nanchang, China) and were in accordance with the ARVO Statement for the Use of Animals in Ophthalmic and Vision Research.

### Resveratrol treatment

**Figure 12:**
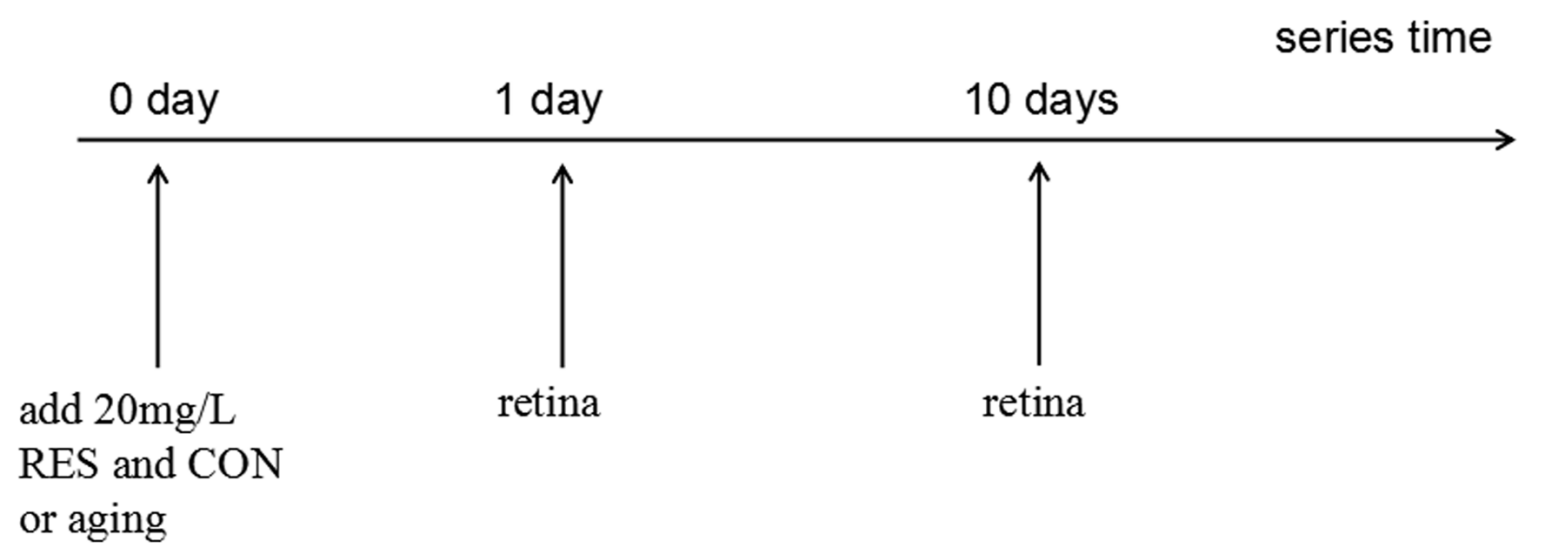
**Resveratrol treatment timeline.** All treatments began at 0 hour and the time points are post-resveratrol administration. CON/aging, 0.04% ethanol; RES, 20mg/L resveratrol + 0.04% ethanol.

The control group was ethanol-control treated zebrafish while the resveratrol group was zebrafish exposed to resveratrol (R5010; Sigma-Aldrich Corp., St. Louis, MO, USA) dissolved in ethanol to the final concentration of 20mg/L for 1 or 10 days. For the resveratrol group, a day of treatment consisted of 10 hours a day under dark condition with resveratrol and another 14 hours under light condition with clean, untreated water [74]. For the control group, the zebrafish were exposed to the same volume of ethanol as the resveratrol group for 1 or 10 days. For immunofluorescence analysis, zebrafish treated for 10 days were used. (Figure 12).

### Quantitative real-time PCR (qRT-PCR)

Total RNA of retinas (n=8 per group) were abstracted with Trizol Reagent (Cat. #92008, Ambion, USA; 15596-018) according to the manufacturer’s instructions. Total RNA was reverse transcribed to cDNA using HiScript® Q RT SuperMix for qPCR (+ gDNA wiper) (Vazyme; R123-01). The cDNA was used as template for qPCR with TB GreenTM Premix Ex TaqTM II (Tli RNaseH Plus) (Takara; RR820A). Specific primers used in the experiment are from PrimerBank and precursor articles (Table 1). The qPCR process, a three steps reaction (95°C denature for 15s, 60°C annealing for 30s and 72°C extension for 24s), was carried out with the StepOne Plus TM Real-time PCR System (Life Technologies, Carlsbad, CA, USA). All data were chosen from the linear phase of amplification for each gene.

**Table 1:** Primers used in the experiment.

Genes	Primers (5’-3’)
Mfn2	F: GCGCCTACATCAAAGAAAGC R: CAGCATCCAAATCCTCATCC
Fis1	F: CTAGCTCCAGGGCCTGTTTGT R: GGTGAAAGGACCCGTTTCCAG
Opa1	F: GCTTGAGCGCTTGGAAAAGGAA R: TGGCAGGTGATCTTGAGTGTTGT
Oma1	F: TGGAGGCGGAAGCGGATCAG R: GAATGAGGCGGTCCAACTGTCTG
Pink1	F: CTGATGACGTTCAGCTGGTG R: CCACAGACTGATGTGCAGGA
Cox2	F:AACTAGGATTCCAAGACGCAGCATC R:AAATAAGAATGATGGCCGGAAGG
IL-1β	F:TCGCCCAGTGCTCCGGCTAC R:GCAGCTGGTCGTATCCGTTTGG
TNF-α	F:AGGAACAAGTGCTTATGAGCCATGC R:AAATGGAAGGCAGCGCCGAG
CuZn-Sod	F:GTCGTCTGGCTTGTGGAGTG R:TGTCAGCGGGCTAGTGCTT
Mn-Sod	F:CCGGACTATGTTAAGGCCATCT R:ACACTCGGTTGCTCTCTTTTCTCT
Gpx	F:AGATGTCATTCCTGCACACG R:AAGGAGAAGCTTCCTCAGCC
Ampk	F:ATCATAGACAACCGCCGCATTA R:TTGGCTCGCCGTACACCA
Sirt1	F:CAAGGAAATCTACCCCGGACAGT R:CAGTGTGTCGATATTCTGCGTGT
Pgc1α	F:CCCCTTTGCCCTGACCTGCCTGAG R:GAAGGACAGCTCTGATCACTGGCATTGG
Beta-actin	F: CCCAAGGCCAACAGGGAAAA R: GGTACGACCGGAGGCATACA
Nuclear chain	F: ATGGGCTGGGCGATAAAATTGG R: ACATGTGCATGTCGCTCCCAAA
Long mitochondrial chain	F: TTAAAGCCCCGAATCCAGGTGAGC R: GAGATGTTCTCGGGTGTGGGATGG
Short mitochondrial chain	F: CAAACACAAGCCTCGCCTGTTTAC R: CACTGACTTGATGGGGGAGACAGT

### Mitochondrial DNA analysis

Total DNA was extracted from retinas using the Ezup column animal genomic DNA extraction kit (B518251; Sangon Biotech, Shanghai, China). Short mitochondrial chain primers and long mitochondrial chain primers [78] (Table 1) were chosen to amplify DNA using Taq DNA Polymerase (Mg2+ plus buffer) (Vazyme; P101). The long mitochondrial chain reaction procedure was 19 cycles (94°C for 15 seconds and 68°C for 12 minutes), and then an extension step (72°C for 10 minutes). The short mitochondrial chain reaction procedure was 25 cycles (94°C for 30 seconds, 62°C for 45 seconds, and 72°C for 30 seconds), and then an extension step (72°C for 10 minutes). All data were chosen from the linear phase of amplification. Amplified DNA were analyzed on a 0.8% agarose gel (with ethidium bromide) and observed by Image Lab under UV light, and were quantified with ImageJ. A DNA Ladder and Low DNA mass ladder (Takara) were used to benchmark the DNA band sizes.

The total DNA extracted from retina was also used for analysis of mitochondrial copy number (mtCN). Short mitochondrial chain and Nuclear chain (Table 1) were chosen to amplify DNA using TB GreenTM Premix Ex TaqTM II (Tli RNaseH Plus) (Takara; RR820A). The reaction procedure was 25 cycles (94°C for 30 seconds, 62°C for 45 seconds, and 72°C for 30 seconds), and then an extension step (72°C for 10 minutes) and was carried out with the StepOne Plus TM Real-time PCR System (Life Technologies, Carlsbad, CA, USA). All data were chosen from the linear phase of amplification for each sequence.

### Western blot analysis

Proteins were extracted from zebrafish retinas (n=10) with RIPA buffer (R0010; Solarbio, Beijing, China) containing Phenylmethanesulfonyl fluoride (PMSF; Solarbio, Beijing, China). The concentration of protein was measured with the BCA Protein Assay Kit (Beyotime Biotechnology, Shanghai, China). Protein samples (10 μg each) were separated by 10% SDS-PAGE and transferred to nitrocellulose membranes. 5% non-fat milk dissolved in Tris-buffered saline containing Tween-20 was used to block the blots before applying primary antibodies for overnight at 4°C (Table 2). Then, peroxidase-conjugated secondary antibodies (goat anti-rabbit and goat anti-mouse (ZSGB-BIO, Beijing, China) were added at 1:2500 for 1 hour at room temp. Protein bands were then observed and analyzed using a SYNGENE imaging system (Cambridge, UK) and ImageJ (National Institutes of Health, Bethesda, MD, USA).

**Table 2:** Primary antibodies used in the experiment.

Antibody	Source	Catalog No.	Type	Dilution	MW(kD)
Mfn2	Abcam	Ab56889	Mouse mAb	1:1000(WB) 1:50(IF)	75
Pink1	Abcam	Ab23707	Rabbit mAb	1:1000(WB) 1:50(IF)	66.50
Opa1	BD	612606	Mouse mAb	1:1000(WB)	80-100
mTOR	CST	#2983	Rabbit mAb	1:1000(WB)	289
P-mTOR	CST	#5536	Rabbit mAb	1:1000(WB) 1:50(IF)	289
AKT	CST	#4691	Rabbit mAb	1:1000(WB)	60
p-AKT-Thr308	CST	#13038	Rabbit mAb	1:1000(WB) 1:50(IF)	60
p-AKT-Ser473	CST	#4060	Rabbit mAb	1:1000(WB)	60
LC3B	CST	#2775	Rabbit mAb	1:1000(WB)	16.14
COX2	Gayman	#160106	Rabbit mAb	1:1000(WB)	75
iNOS	Invitrogen	PAI-036	Rabbit mAb	1:1000(WB)	130
Beta-actin	TRANS	HC201	Mouse mAb	1:1000(WB)	42
Beta-tubulin	TRANS	J10715	Donkey anti-mouse	1:1000(WB)	55
					

### Immunofluorescence

Zebrafish were first anesthetized in ice water and then the eyeballs were carefully removed and fixed in 4% PFA overnight. After fixation, eyes were transferred to an EP tube for dehydration in 10%, 20%, and 30% sucrose solution until the eyes settled to the bottom in each sucrose concentration. Each eye was then solidified with OCT (optim alcutting tem perature) under -80°C for conservation or -20°C in a freezing microtome for slicing. The microtome maintained the temperature between -22°C to -20°C, and frozen eyes were sliced into 5μm thick slices. Slices were washed with 1×PBS 3×5 minutes. Water surrounding the tissue was blot up and circles were drawn on each tissue using a fluorescent pen. Retina slices were then blocked in PBS containing 0.3% BSA (Solarbio, Beijing, China) for an hour at room temperature and washed with 1×PBS 3x5 minutes before incubating with primary antibodies (Table 2) overnight at 4°C. Tissue slices incubated in PBS without primary antibodies were used as negative controls, and rat tissue slices incubated in PBS with same primary antibodies were used as positive controls. 1× TBST washing for 3x5 minutes was performed before and after incubation with Alexa Fluor 488 anti-mouse (H+L) or Alexa Fluor 488 anti-rabbit (H+L) secondary antibodies (dilution, 1:200; Life Technologies, Eugene, OR, USA) for one hour at room temperature. DAPI (Boster, Wuhan, China) staining of cellular nuclei was then performed prior to covering the slides with coverslips. Finally, retina slices were observed by confocal microscopy (OLYMPUS IX71, Japan).

### Data analysis

Data were analyzed with GraphPad Prism 7.0 and ImageJ, and the error bars are mean ± SEM. Significance was checked by ANOVA followed by unpaired t-test, n ≥ 3. P < 0.05 was considered as significant.
